# *Toxoplasma gondii* Recruits Factor H and C4b-Binding Protein to Mediate Resistance to Serum Killing and Promote Parasite Persistence *in vivo*

**DOI:** 10.3389/fimmu.2019.03105

**Published:** 2020-01-17

**Authors:** Patricia M. Sikorski, Alessandra G. Commodaro, Michael E. Grigg

**Affiliations:** ^1^Molecular Parasitology Section, Laboratory of Parasitic Diseases, National Institute of Allergy and Infectious Diseases, National Institutes of Health, Bethesda, MD, United States; ^2^Department of Microbiology and Immunology, Georgetown University Medical Centre, Georgetown University, Washington, DC, United States

**Keywords:** complement system resistance, *Toxoplasma gondii*, alternative pathway regulation, immune evasion, protozoan parasites

## Abstract

Regulating complement is an important step in the establishment of infection by microbial pathogens. *Toxoplasma gondii* actively resists complement-mediated killing in non-immune human serum (NHS) by inactivating C3b, however the precise molecular basis is unknown. Here, a flow cytometry-based C3b binding assay demonstrated that Type II strains had significantly higher levels of surface-bound C3b than Type I strains. However, both strains efficiently inactivated C3b and were equally resistant to serum killing, suggesting that resistance is not strain-dependent. *Toxoplasma* activated both the lectin (LP) and alternative (AP) pathways, and the deposition of C3b was both strain and lectin-dependent. A flow cytometry-based lectin binding assay identified strain-specific differences in the level and heterogeneity of surface glycans detected. Specifically, increased lectin-binding by Type II strains correlated with higher levels of the LP recognition receptor mannose binding lectin (MBL). Western blot analyses demonstrated that *Toxoplasma* recruits both classical pathway (CP) and LP regulator C4b-binding proteins (C4BP) and AP regulator Factor H (FH) to the parasite surface to inactivate bound C3b–iC3b and C3dg and limit formation of the C5b-9 attack complex. Blocking FH and C4BP contributed to increased C5b-9 formation *in vitro*. However, parasite susceptibility *in vitro* was only impacted when FH was blocked, indicating that down regulation of the alternative pathway by FH may be more critical for parasite resistance. Infection of C3 deficient mice led to uncontrolled parasite growth, acute mortality, and reduced antibody production, indicating that both the presence of C3, and the ability of the parasite to inactivate C3, was protective. Taken together, our results establish that *Toxoplasma* regulation of the complement system renders mice resistant to acute infection by limiting parasite proliferation *in vivo*, but susceptible to chronic infection, with all mice developing transmissible cysts to maintain its life cycle.

## Introduction

At early stages of infection, intracellular protozoan parasites are vulnerable to humoral attack in the extracellular environment. The complement system, a humoral effector system that activates on pathogen surfaces, is considered the first line of defense. Complement activation is initiated by three pathways, known as the classical, lectin, and alternative pathways. Recognition of immune complexes and microbial carbohydrates activate the classical (CP) and lectin (LP) pathways, respectively. The alternative pathway (AP) is activated by the spontaneous hydrolysis of C3 and can further activate on non-self surfaces that lack host regulator proteins. Recognition triggers a sequential cascade of proteolytic events that results in the assembly of complexes known as convertases, that generate active proteins C3a and C3b, and ultimately culminates in the formation a pore complex to mediates direct lysis of pathogens. Considering the vast majority of invading pathogens have mechanisms to protect from complement attack, the complement system has evolved to overcome pathogen evasion of serum killing by employing several lysis-independent effector functions that depend on C3a and C3b to enhance innate and adaptive immunity, such as promoting inflammation, targeting pathogens for phagocytosis, and stimulating adaptive immunity (1). Excessive complement activation, however, can also result in damage and inflammation in nearby tissues, thus it must be tightly regulated by the host (2).

Complement regulation in the host, and for many pathogens, is a complex interaction that requires multiple factors. Several intracellular protozoan parasites including *Toxoplasma, Leishmania, Plasmodium*, and *Trypanosoma*, have evolved efficient subversion strategies to evade complement function. Each of these parasites target C3 convertases and the activation product C3b to impede direct killing (3–9). Several surface-expressed parasite molecules that functionally mimic or sequester host complement regulatory proteins have been identified in trypanosomatids (gp160, LPG, gp63) and *Plasmodium spp*. (Pfs47, Pf92) (10–13). To date, however, only one study has shown that *T. gondii* is resistant to complement killing in non-immune serum by inactivating C3b (7), but the mechanism of C3b inactivation remains enigmatic.

*Toxoplasma gondii* is a highly prevalent protozoan parasite that can infect essentially any cell in all mammals, including humans. *Toxoplasma* is comprised of several genotypically variant strains that have been shown to differ in their virulence across a wide range of hosts (14–22). Type II strains are most prevalent in human and animal infections in North America and Europe (15, 23, 24). Less frequently, human infection with Type I strains or atypical strains with Type I alleles have been associated with causing encephalitis in HIV patients (25) or recurrent ocular disease in otherwise healthy people (26). In order to establish infection and cause disease in a large number of hosts, *T. gondii* employs large families of polymorphic effector proteins to modulate host immune responses. Murine studies have identified several polymorphic secreted effector proteins, including rhoptry, and dense granule proteins, that manipulate intracellular immune signaling (27–29). However, the *T. gondii* factors orchestrating resistance to host defenses during the parasite's extracellular phase, including the complement system, are still poorly characterized.

Since the initial study done almost 30 years ago, no further studies have been done to identify the factors mediating *T. gondii* complement resistance. Here, we used both *in vitro* and *in vivo* studies to better understand the significance of parasite mediated regulation of the complement system. We developed flow cytometry-based assays to measure complement activation on the parasite surface to improve our understanding of this interaction. Using this assay, strain specific differences in C3b deposition between *T. gondii* Type I and Type II strains were observed. However, the higher levels of C3b on Type II strains did not impact parasite susceptibility to serum killing. The ability of *Toxoplasma* to recruit both C4b-binding protein (C4BP) and Factor H (FH) to parasite surfaces suggested a role for lectin and alternative pathways in complement activation. Both regulators contributed to regulation of C5b-9 formation, however Factor H recruitment may ultimately be the critical factor to resist lysis. Mice rendered C3 deficient were highly susceptible to acute infection with *T. gondii*, indicating that complement is necessary for resistance to acute mortality, and that parasites hijack host regulators of the complement cascade to ensure mice are likewise susceptible to a chronic transmissible infection for this highly successful protozoan parasite.

## Materials and Methods

### Parasites

*Toxoplasma gondii* tachyzoites were maintained by *in vitro* passaging in confluent monolayers of human foreskin fibroblasts (HFF) in Dulbecco modified Eagle medium (DMEM) with 10% fetal bovine serum (FBS), 2 mM glutamine, penicillin, streptomycin, and 10 μg/ml gentamicin at 37°C, 5% CO_2_. Parasites were counted using a hemocytometer. Type II strains ME49 (ME49, California sheep) and CZ1 (Czech Republic tiger) and Type I strain RH (ERP) were used in subsequent assays.

### Flow Cytometry to Determine C3b Deposition, C1q and MBL Binding, Lectin Binding Assay, and Parasite Viability

Parasites were collected from a freshly lysed monolayer of HFF cells, filtered, and washed twice with PBS to remove media. For time course studies, 1 × 10^6^ parasites (per time point) were incubated with 10% non-immune human serum (pooled NHS, Cedarlane; MBL deficient serum, BioPorto) in complement activating buffer HBSS^++^ (Hanks Buffered Saline Solution, 1 mM MgCl_2_, 0.15 mM CaCl_2_). All serum was tested by IFA and flow cytometry for the presence or absence of human anti-*Toxoplasma* IgG prior to use. Complement activation was stopped using cold PBS and washed two times with cold PBS to remove excess serum. For viability assays, parasites were exposed to 10%, 20, 40, and 60% NHS for 60 min at 37°C, washed three times with cold PBS to remove excess serum, and stained with a fixable viability stain (Live/Dead fixable violet stain, Thermo Fisher) for 15 min. Parasites were then fixed with 1% PFA solution for 10 min, washed, and stained with: mouse α-human C3b/iC3b monoclonal antibody (clone 7C12) 1:500 (Cedarlane), goat α-human MBL 1:1,000 (R&D Systems), mouse α-human C1q (clone MhC5B9) 1:250 (Cedarlane) or mouse α-human C5b-9 (clone aE11) neo-epitope 1:500 (Santa Cruz Biotechnology), followed by α-mouse APC (eBiosciences), α-goat APC conjugated antibody (Jackson Laboratories), or streptavidin-APC, all at 1:1,000. For lectin binding assays, 5 ug/ml of biotinylated lectins (Vector Labs) were incubated with parasites for 1 h on ice, washed, and incubated with streptavidin-APC (1:1,000). 20,000 events were collected on a BD Fortessa flow cytometer and analyzed by FACs DIVA, FlowJo and Prism software.

### Western Blotting

Parasites were treated with 10% NHS for 0–60 min at 37°C, washed three times with cold PBS, lysed with 1% NP-40 lysis buffer (1% NP-40, 150 mM NaCl, 50 mM Tris pH 8.0) for 1 h on ice, and pelleted at 14,000 rpm for 20 min to remove insoluble material. Lysates were prepared with 2X Laemmli sample buffer (Bio-Rad) with 5% 2-mercaptoethanol and boiled for 5 min at 95°C. Samples were loaded onto 12% polyacrylamide gels (Bio-Rad) and transferred onto nitrocellulose using GenScript eBlot system. Membranes were blocked for 1 h with 5% non-fat dry milk in PBS + 0.05% Tween-20. The following antibody dilutions were used: rabbit anit-SRS29B (formerly SAG1, 30 kDa) 1:5,000, rabbit anti-C4BP (alpha chain, 70 kDa) (AssayPro) 1:1,000, goat anti-C3 (C3, C3b, iC3b) and goat anti-Factor H (full length protein, 155 kDa) antibodies (CompTech) were both used at 1:20,0000, anti-rabbit HRP 1:10,000 (Sigma), and anti-goat HRP 1:5,000 (Santa Cruz Biotechnology, Inc.). Proteins were detected with Clarity Western ECL Substrate (Bio-Rad).

### C4BP and Factor H Blocking

10% NHS in HBSS^++^ was incubated with 1:100 or 1:400 dilution of polyclonal goat α-human Factor H (FH) or rabbit α-human C4b-binding protein (C4BP) for 1 h on ice, and then added to 1 × 10^6^ parasites and incubated at 37°C for 60 min. Parasites were then stained with viability dye and α-mouse C5b-9 as described above and analyzed by flow cytometry.

### Mouse Infection

Eight-week-old C57BL/6J mice and C3^−/−^ were purchased from Jackson Laboratories. C3 deficient mice were reportedly backcrossed to C57BL/6 mice for at least 5 generations prior to sending to the Jackson Laboratory Repository. F2 homozygous C57BL/6J C3^−/−^ mice and F2 heterozygous mice were generated by crossing F1 progeny from a C57BL/6J WT × C3^−/−^ cross with a homozygous C3^−/−^ mouse. Mice were infected intraperitoneally (i.p.) with tachyzoites or tissue cysts. Parasites cultured in HFFs cells were washed in twice in PBS by centrifugation at 1,250 rpm for 10 min and counted on a hemocytometer. Mice were injected with 2 × 10^3^ tachyzoites in 500 μl in incomplete RPMI for infections with CZ1 Type II strains. ME49 type II strain of *T. gondii* was used for production of tissue cysts in C57BL/6 mice. Tissue cysts used in experiments were obtained from mice that were inoculated 1–3 months previously, with forty cysts by gavage. Animals were sacrificed, and the brains were removed and homogenized in 1 ml of phosphate buffer saline (PBS) (pH 7.2). Tissue cysts were counted on the basis of 3 aliquots of 20 μl and a total or 40 cysts in 300 ul of PBS were used to infect mice using oral gavage.

### Plaque Assays to Quantify Parasite Load

Mice were euthanized at day 7 post infection. Peritoneal fluid was collected by injecting 5 ml of sterile PBS into the peritoneal cavity of euthanized mice. Spleens, lungs, brains, liver, and thymus were harvested and homogenized over 70-μm strainers in 5 ml of RPMI media. Ten-fold serial dilutions starting with 500 μl of resuspended homogenate were added to HFF monolayers in 12 well-plates and cultured undisturbed in DMEM media for 7–10 days at 37°C. HFFs were fixed in methanol and stained with crystal violet. Plaques were counted using a Zeiss axiovert 40c inverted microscope and parasite loads were expressed as plaque forming units (PFU) per organ.

### ELISA for IgG Quantification

Fifty microliters of 0.5 μg of total *Toxoplasma* ME49 lysate was loaded into each well of a polystyrene ELISA plate (Falcon flat-bottom ELISA plate) for coating overnight at 4°C. Wells were then blocked with 200 μL of a 2% casein solution in PBS with 0.01% thimerosal for 2 h at room temperature. Fifty microlitre of diluted infection serum (1:40) was then added to each well, and plates were incubated at room temperature for 3 h. ELISA plates were washed 4 times with 0.1% Tween-20 in PBS before incubation with horseradish peroxidase-conjugated detection antibody against mouse IgG, IgG2a, and IgG2b (Sigma, 1:1,000). After incubation at room temperature for 1 h, the secondary antibodies were aspirated, and the plates were washed with 0.1% Tween-20 four times and with PBS twice. Plates were developed with 150 μL of the ABTS reagent (Kirkegaard and Perry Laboratories), and the absorbance was measured at 405 nm after 20 min.

### Statistical Analysis

Statistical significance analyses were conducted in Graph Prism Version 8.2.0. FACs analyses was expressed as mean ± SEM (standard error of the mean) of three independently performed experiments and data were compared by multiple Student t tests with Holm-Sidak *post hoc* corrections for multiple comparisons. Survival rates were compared by log-rank survival analysis of Kaplan-Meier curves. Analyses of parasite burden and IgG levels from *in vivo* experiments are expressed as mean ± SEM from two independently performed experiments and compared by unpaired Student's *t* test. Mean differences, which are denoted by an asterisk, were considered statistically significant if ^*^p < 0.05, ^**^p < 0.005, ^***^p < 0.001, ^****^p < 0.0001.

## Results

### Parasite Genotype Affects C3b Deposition

**Figure 1:**
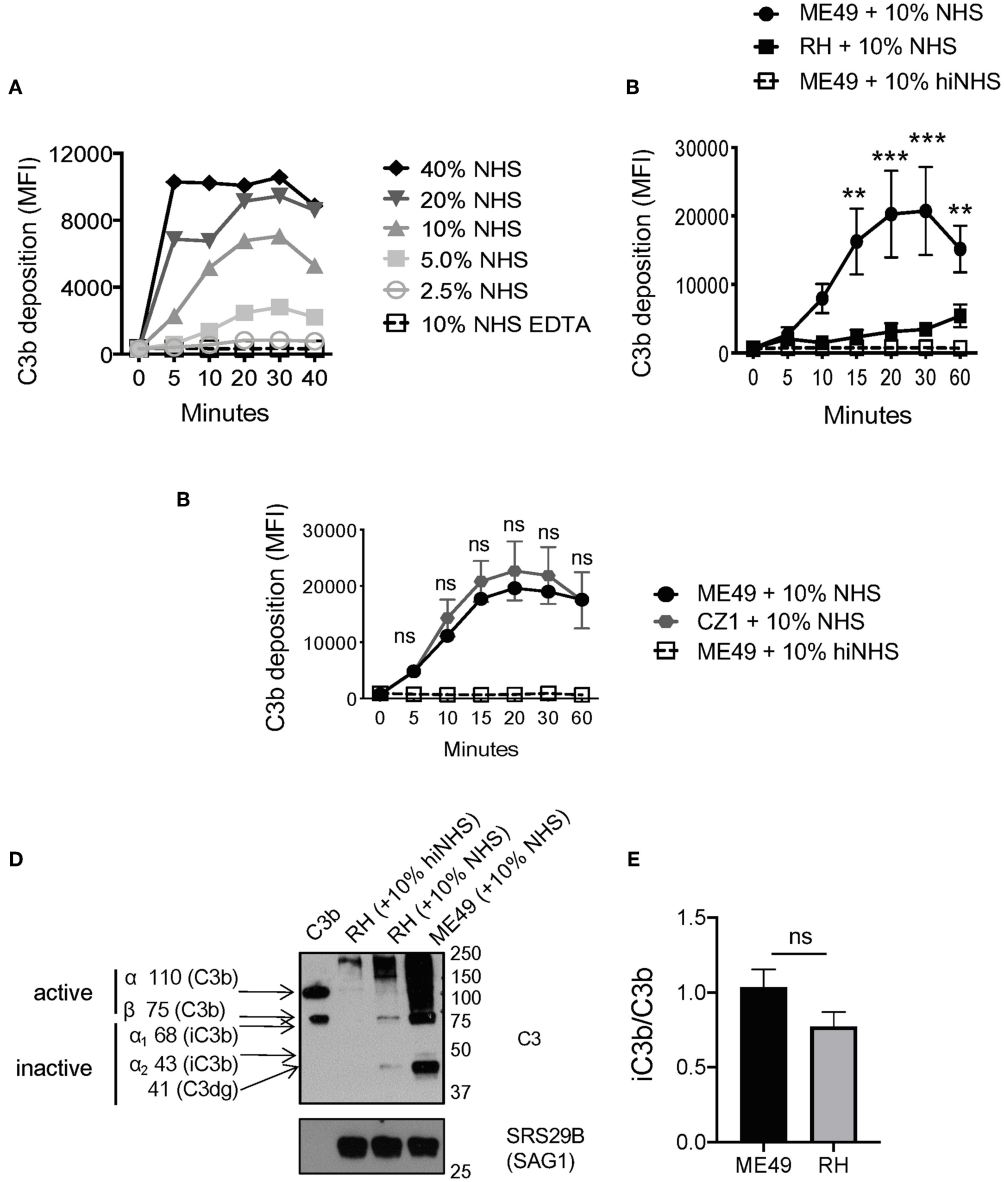
Strain-specific difference in C3b deposition. **(A)** Titration of NHS. Type II ME49 parasite were incubated in non-immune human serum (NHS) serially diluted 2-fold, from 40 to 2.5%. Dilutions were made in Hanks Buffered Saline Solution supplemented with 0.15 mM CaCl_2_ and 1 mM MgCl_2_ to facilitate classical/lectin and alternative pathway activation, respectively (HBSS^++^). Parasites were stained with a mouse monoclonal α-human C3b/iC3b antibody, 1:500 (Cedarlane). 10 mM EDTA was used as a negative control to inhibit all complement pathways. C3b deposition was measured by flow cytometry. Flow cytometry data are shown as mean ±SEM from three independently performed experiments. **(B)** 1 × 10^6^ Type II ME49 (closed circle) and 1 × 10^6^ Type I RH (closed square) parasites were incubated in 10% NHS in HBSS^++^ over 60 min and C3b deposition was measured using flow cytometry as described above. ME49 parasites incubated with 10% heat inactivated serum (hiNHS) was used as a negative control (open square, dotted line). Flow cytometry data are shown as mean ±SEM from five independently performed experiments and statistically significant differences between Type I and Type II strains were determined using multiple Student's *t* test with Holm-Sidak correction for multiple comparisons, **p < 0.005, ***p < 0.001. **(C)** Flow cytometry results from C3b deposition time course comparing Type II strains ME49 (black) and CZ1 (gray) in 10% NHS over 60 min. Flow cytometry data are shown as mean ±SEM from three independently performed experiments; no significant differences between Type II strains CZ1 and ME49 using multiple Student's *t* test with Holm-Sidak correction for multiple comparisons, ns = not significant. **(D)** Western blots showing the difference in levels of C3b between 1 × 10^6^ Type II ME49 vs. 1 × 10^6^ RH parasites incubated 20' in 10% NHS at 37°C **(E)** iC3b/C3b ratio for ME49 and RH incubated in 10% NHS for 20 min. The ratio was determined by quantifying the density of active C3b (using the 75 kDa beta chain of C3b) and inactive iC3b and C3dg (43 and 41 kDa bands, respectively) from three independent western blot images using Image J software. There was no significant difference between the ratio of iC3b/C3b between ME49 and RH using an unpaired Student's *t* test, ns, not significant.

Previous work showed that C3b was deposited and inactivated on the surface of *Toxoplasma* Type I and Type II strains (7). To confirm this finding, several new assays were developed to more precisely measure complement activation and resistance. Previously, radiolabeled C3 was added to non-immune human serum (NHS) to detect levels of C3b that bound the parasite surface upon activation (7). C3 is the most abundant complement protein in human serum (~1.0 mg/ml), thus rapid saturation on the parasite surface may impact the sensitivity of measuring C3b deposition. Therefore, we developed a flow cytometry-based assay using monoclonal antibodies specific for human C3b (Supplemental Figures 1A,B). Parasites were incubated in serially diluted serum (2-fold dilutions from 40% NHS-2.5% NHS) to determine the optimal concentration that gave good sensitivity without rapid saturation. Whereas, 20 and 40% NHS rapidly saturated within minutes, and 5 and 2.5% were less sensitive in the absolute level of C3b detected, we found that 10% NHS was the optimal concentration, it did not saturate in minutes and was still sensitive enough to detect variable levels of C3b deposited depending on the conditions used (Figure 1A).

The level of C3b deposition using 10% NHS was determined for two *T. gondii* strains, Type I strain RH and Type II strain ME49. Contrary to previous work (7), our method identified that Type II strains are coated in a time-dependent manner with significantly higher levels of C3b than Type I strains (Figure 1B and Supplemental Figures 2A,B). Increased deposition of C3b on the surface of Type II strains was a general trait, as equivalent levels and kinetics of C3b were found on Type II strains derived from different animal hosts (ME49, California sheep) and different geographies (CZ, Czech Republic tiger) (Figure 1C).

### *T. gondii* Inactivation of C3b and Serum Resistance Is Strain Independent

**Figure 2:**
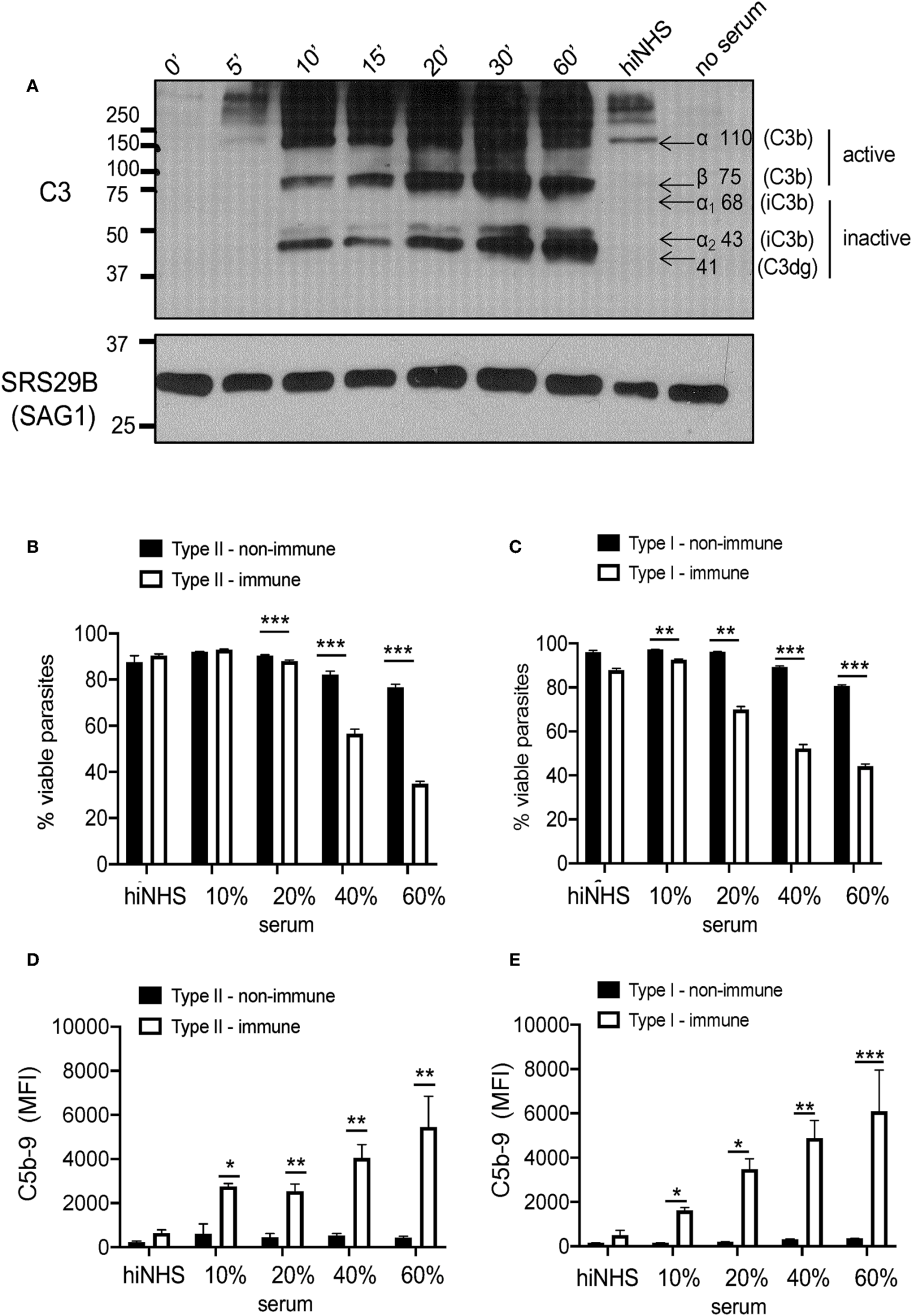
Serum resistance is strain independent. **(A)** Kinetics of C3b inactivation on Type II ME49 parasites (5 × 10^6^) over 60 min in 10% NHS probed for C3 and its catabolites using a goat anti-human C3 polyclonal antibody 1:20,000 (CompTech) and donkey anti-goat 1:5,000 (Santa Cruz Biotechnology). Arrows correspond to sizes of C3 (active C3b α chain 110 kDa, β chain 75 kDa; inactive iC3b α_1_ 68 & α_2_ 43 kDa, and C3dg 41 kDa). SRS29B (formerly SAG1, 1:5,000) was used as loading control. Western blot image shown is representative of at least three independent experiments with similar results. Parasite viability and C5b-9 pore formation was determined by exposing parasites to 10, 20, 40, 60% non-immune (black bars) or immune (open bars) serum for 60 min at 37°C. 20% heat inactivated (hiNHS) non-immune serum was used a negative control. Flow cytometry was used to measure parasite susceptibility by staining Type II **(B)** and Type I **(C)** parasites with a fixable viability stain (Life Technologies) and membrane attack complex formation **(D,E)** using monoclonal mouse α-human C5b-9 (Santa Cruz Biotechnology, 1:500). Immune serum was used as a positive control for parasite lysis due to classical pathway activation. Flow cytometry data are shown as mean ± SEM from three independently performed experiments. Statistically significant differences between parasites incubated in immune vs. non-immune serum were determined using multiple Student's *t* test with Holm-Sidak correction for multiple comparisons, *p < 0.05, **p < 0.01, ***p < 0.001.

One limitation of this flow cytometric assay is that it cannot distinguish between active vs. inactive forms of C3b. Western blot studies were thus performed to assess the form of C3b covalently associated with parasite surfaces. Both Type I and Type II strains inactivated C3b (Figure 1D) in a time dependent manner (Supplemental Figures 2A,B). The ratio of iC3b/C3b was not statistically different between Type I and Type II strains despite their overall differences in the level of C3b deposited on each parasite surface (Figure 1E), suggesting that C3b inactivation is strain independent. Western blot analysis showed that C3b is inactivated within 10 min and the degradation pattern of C3b was consistent with host mediated inactivation by co-factor Factor H and serine protease Factor I (active C3b α 110 kDa, β 75 kDa; inactive iC3b 68 & 43kDa, C3dg 41kD) (Figure 2A). Taken together, although there are significant differences in the quantity of C3b between Type I and Type II strains, there is no absolute difference in the inactivation of C3b between strain types.

To determine whether the inactivation of C3b was sufficient to obviate attack complex formation (C5b-9) to prevent serum killing, parasite viability was evaluated using increasing amounts of NHS. Previous work measured viability of parasites exposed to serum using a relatively insensitive trypan blue exclusion test (7). Here, we utilized a fixable amine-reactive viability dye to measure resistance to serum killing by flow cytometry as well as a monoclonal anti-human C5b-9 (membrane attack complex) antibody to measure viability and pore formation, respectively. Despite differences in the absolute levels of C3b, Type I and Type II strains remained equally viable in the presence of non-immune serum, independent of the serum concentration used (Figures 2B,C), As a positive control for complement meditated lysis, parasites were incubated in the presence of immune serum, in which parasites are susceptible to classical pathway activation (30), and both strains showed a similar loss of viability and susceptibility to lysis in 20, 40, and 60% immune serum (Figures 2B,C). C5b-9 membrane attack complex formation was dependent on the immune status and concentration of serum used, but was independent of strain type (Figures 2D,E). Taken together, our data demonstrate that both Type I and Type II strains inactivate C3b, regardless of the C3b levels deposited, and this was sufficient to prevent serum killing and C5b-9 formation in non-immune human serum.

### Complement Activation on *T. gondii* Is Ca^2+^ Dependent

**Figure 3:**
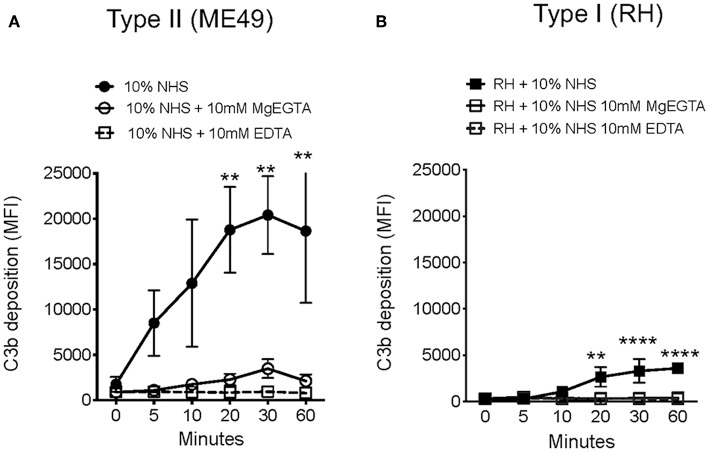
*Toxoplasma gondii* activates Ca^2+^ dependent complement pathways. Flow cytometry results of C3b deposition time course over 60 min at 37°C on Type II ME49 parasites **(A)** and Type I RH parasites **(B)** incubated in 10% NHS in HBSS^++^ or with HBSS^++^ treated with 10 mM MgEGTA to inactivate CP and LP. Ten millimolar EDTA was added to 10% NHS to inactivate all complement pathways (open square, dotted line). Flow cytometry data are shown as mean ± SEM from three independently performed experiments. Significant differences in C3b deposition between parasites exposed to NHS and NHS treated with 10 mM MgEGTA was compared using multiple Student's *t* test with Holm-Sidak correction for multiple comparisons, **p < 0.01, ****p < 0.0001.

Previous work showed that the alternative pathway was a major contributor to C3b deposition on *T. gondii* (7), but this published work preceded the discovery of the lectin pathway. To determine the contributions of the classical, lectin and alternative pathways in the deposition and inactivation of C3b deposited on *Toxoplasma*, parasites were incubated in 10% NHS plus 10 mM MgEGTA in order to inactivate the Ca^2+^-dependent classical and lectin pathways, thus any C3b deposition measured on Type II ME49 parasites was due to AP activation. Contrary to previous findings (7), these results showed AP activation contributed little to the initial C3b deposition on both Type I and Type II strains, indicating that Ca^2+^-dependent complement activation was the major contributor to C3 deposition (Figures 3A,B). Ca^2+^ dependent CP and LP activation suggests that *Toxoplasma* harbors surface carbohydrates that can be specifically recognized by pattern recognition molecules of the CP and LP pathways to promote complement activation. To determine if the classical pathway pattern recognition molecule C1q bound *T. gondii*, C1q binding was assessed by flow cytometry in both immune and non-immune serum. C1q bound *T. gondii* in the presence of immune serum, as expected, but there was no evidence of C1q binding in non-immune serum (Supplemental Figure 3).

**Figure 4:**
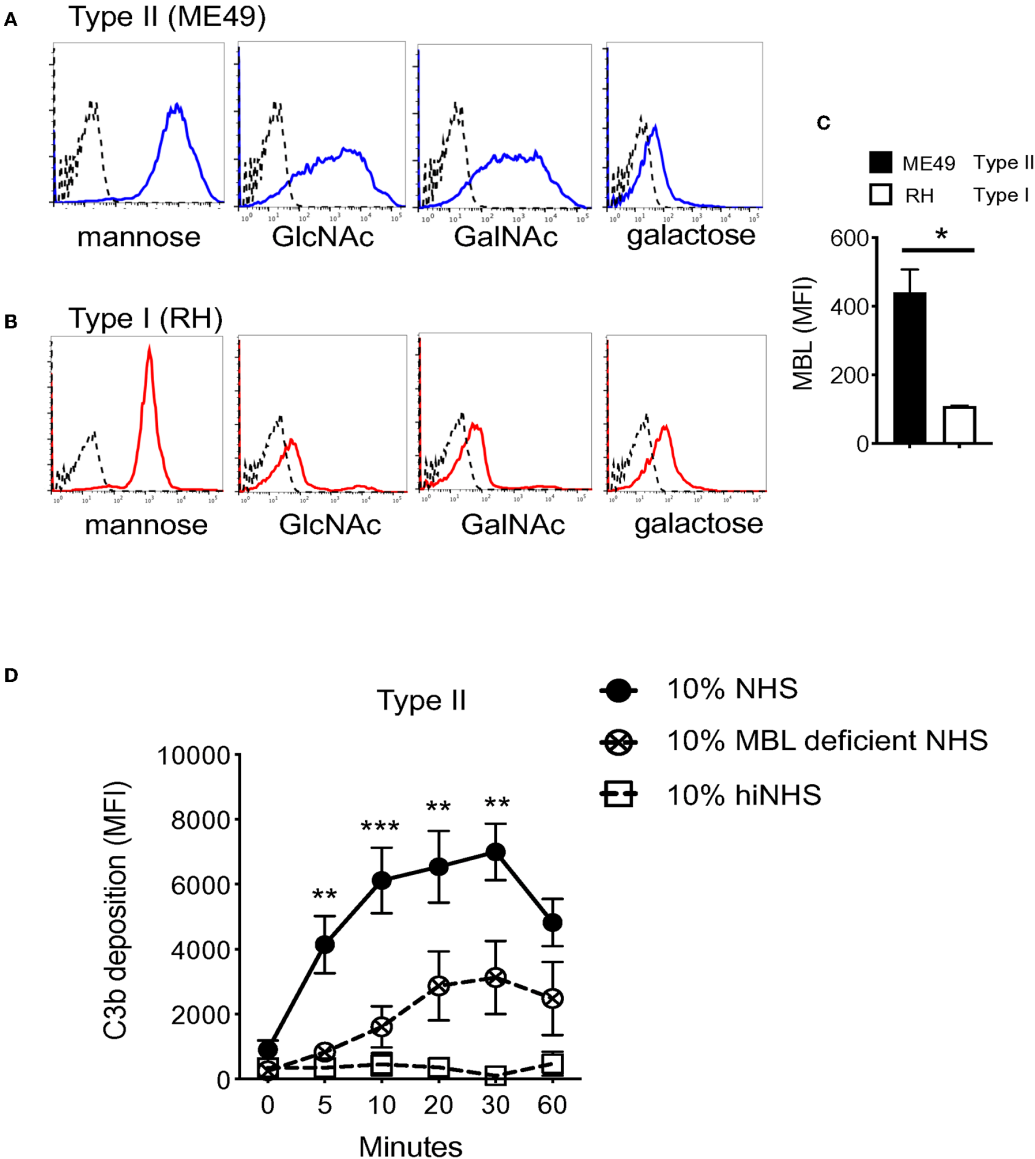
Heterogeneity in surface carbohydrate composition between Type I and Type II strains affects MBL recognition. Carbohydrate ligands on the parasite surface were surveyed using a panel of biotinylated lectins with known specificities (Vector Laboratories, α-linked mannose/α-linked glucose (ConA Concanavalin A), N-Acetylglucosamine, GlcNac (WGA, wheat germ agglutinin), galactose (RCA, *Ricinus communis* agglutinin I) and, N-Acetylgalactosamine, GalNAc (DBA, *Dolichos biflorus* agglutinin). 1 × 10^6^ parasites were incubated with 5 μg/ml of biotinylated lectin for 1 h on ice, followed by streptavidin-APC (1:1,000) for 30 min, and analyzed by flow cytometry. Representative histograms of lectin binding between Type II (ME49, blue) **(A)** and Type I (RH, red) **(B)** compared to unstained control (black, dotted line). **(C)** Flow cytometry results of MBL (mannose binding lectin) binding to Type II (ME49, black bar) and Type I (RH, open bar) parasites incubated in 10% NHS for 60' on ice. MBL was detected using goat α- human MBL (R&D Systems 1:1,000) followed by anti-goat-APC (1:1,000). Flow cytometry data are shown as mean ±SEM from three independently performed experiments. Significant differences in MBL binding between Type I RH and Type II ME49 was determined using an unpaired *t* test, *p < 0.05. **(D)** Type II ME49 parasites were incubated in 10% serum from a patient with a mutation in the human MBL gene (open circle, dotted line), 10% NHS from a MBL sufficient single donor in HBSS^++^ (solid circle, solid line) or 10% heat inactivated NHS (hiNHS) in HBSS^++^ (open square, solid line). Flow cytometry data are shown as mean ± SEM from three independently performed experiments. Significant differences in C3b deposition between parasites exposed to MBL sufficient NHS and MBL deficient NHS was compared using multiple Student's *t* test with Holm-Sidak correction for multiple comparisons, **p < 0.01, ***p < 0.001.

LP recognition of carbohydrates on microbial surfaces is mediated by two families of lectin proteins, a subfamily of C-type lectins which include mannose binding lectin (MBL), and ficolins. MBL recognizes terminal monosaccharides such as glucose, mannose, or N-acetylglucosamine (GlcNAc) in a Ca^2+^-dependent manner (31, 32). Ficolins are similar in structure and bind acetylated compounds such as GlcNAc and N-acetylgalactosamine (GalNAc) (33). Studies have shown that several *T. gondii* proteins important for host-pathogen interactions are N-linked, including rhoptry proteins, one unidentified surface protein p23, and moving junction proteins (34–36). Here, a panel of lectins for known LP activating carbohydrates was used to assess the presence of glycans on the parasite surface using flow cytometry. A strain-specific difference was identified for the binding of ConA, Wheat germ agglutinin (WGA), and *Ricinus communis* agglutinin I (RCA), lectins specific for mannose, GlcNAc, and GalNAc respectively, but not for *Dolichos biflorus* agglutinin (DBA), which binds galactose residues. Specifically, Type II strains bound more lectin than Type I strains (Figures 4A,B). The difference in lectin binding also correlated with surface levels of C3b detected on Type I vs. Type II strains. To evaluate whether such differences in parasite surface carbohydrates influenced LP recognition, binding of MBL on Type I (RH) and Type II strains (ME49) was measured by flow cytometry. Accordingly, the increased levels of carbohydrates observed on Type II strains correlated with significantly higher levels of MBL compared with Type I strains (Figure 4C).

To confirm a role for MBL in LP-mediated C3b activation on the tachyzoite surface, human serum from an individual with a mutation in the MBL gene was tested. This mutation prevents MBL oligomerization required for carbohydrate recognition, and subsequently impairs LP-mediated complement activation (37). When ME49 parasites were incubated in serum from a patient with a mutation in the human MBL gene that prevents MBL oligomerization and thus lectin pathway activation, a significant reduction in C3b deposition was observed compared to normal human serum from a single donor with functional MBL (Figure 4D). This reduction was not as complete as the control MgEGTA treatment, indicating that other homologous carbohydrate recognizing molecules such as ficolins are likely involved in the activation of the LP.

### *Toxoplasma* Recruits Soluble Regulators C4BP and FH to the Parasite Surface

**Figure 5:**
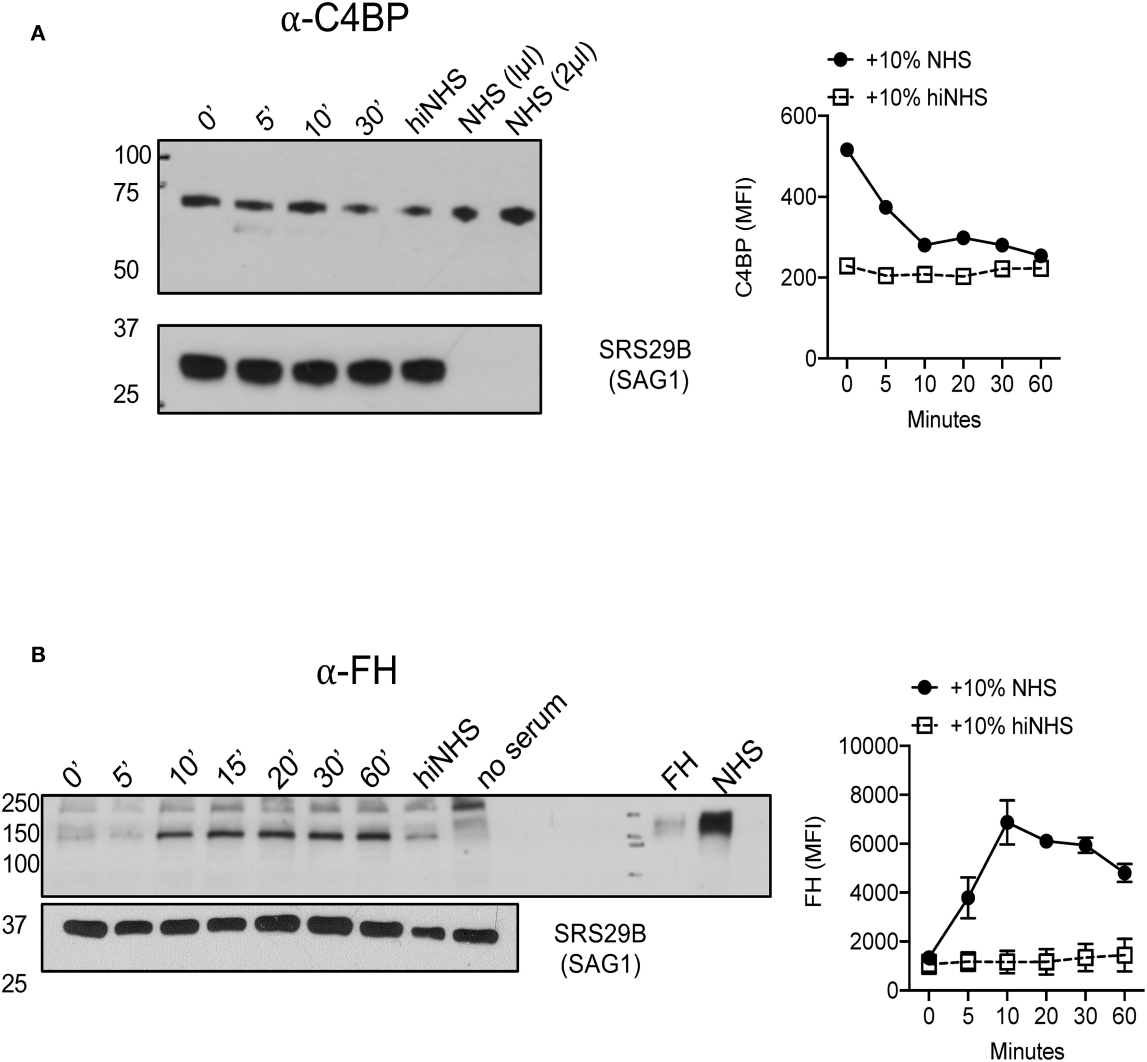
*Toxoplasma gondii* recruits AP regulator Factor H and CP/LP regulator C4b-binding protein to the parasite surface. 1 × 10^6^ Type II ME49 parasites were incubated in 10% NHS for 0–60 min at 37°C. Western blots (left panels) of C4BP **(A)** (rabbit α-human C4BP, AssayPro 1:500) and FH **(B)** (goat α-human Factor H, CompTech 1:20,000) binding. Serum or purified protein was used as a positive control and heat inactivated serum (hiNHS) was used as a negative control. Blots were stripped and re-probed with anti-SRS29B (SAG1) for loading control. Images are from one representative of three independent experiments with similar results. Right panels **(A,B)** represent flow cytometric assays of C4BP **(A)** and FH **(B)** binding to the parasite surface for 0–60 min. Heat inactivated NHS (hiNHS) serum was used as a negative control. Flow cytometry data are shown as mean ± SEM from three independently performed experiments.

Two soluble host proteins that regulate complement activation in non-immune serum are classical and lectin pathway (LP) regulator C4b-binding protein (C4BP) and alternative pathway (AP) regulator Factor H (FH). C4BP primarily targets CP and LP C3 convertases to prevent further cleavage of C3 into its activation products C3a and C3b, however it is also a co-factor for Factor I-mediated cleavage of C4b and C3b (38, 39). FH regulates AP activation by facilitating the decay of AP C3 convertases but also acts as a co-factor for Factor I-mediated inactivation of C3b into iC3b and C3dg (40). Previous studies have shown that *Plasmodium falciparum* 6-CYS protein Pf92 regulates complement by recruiting AP regulator FH (13). To investigate whether *Toxoplasma* utilizes a similar strategy to down regulate complement activation, the binding of these host soluble regulators was evaluated. Parasites incubated in 10% NHS recruited both C4BP and FH to the parasite surface by Western blot analyses (Figures 5A,B). These data suggested that *Toxoplasma* specifically recruits regulators of all complement pathways.

### Factor H but Not C4BP Enhances Serum Resistance by Regulating the Alternative Pathway

**Figure 6:**
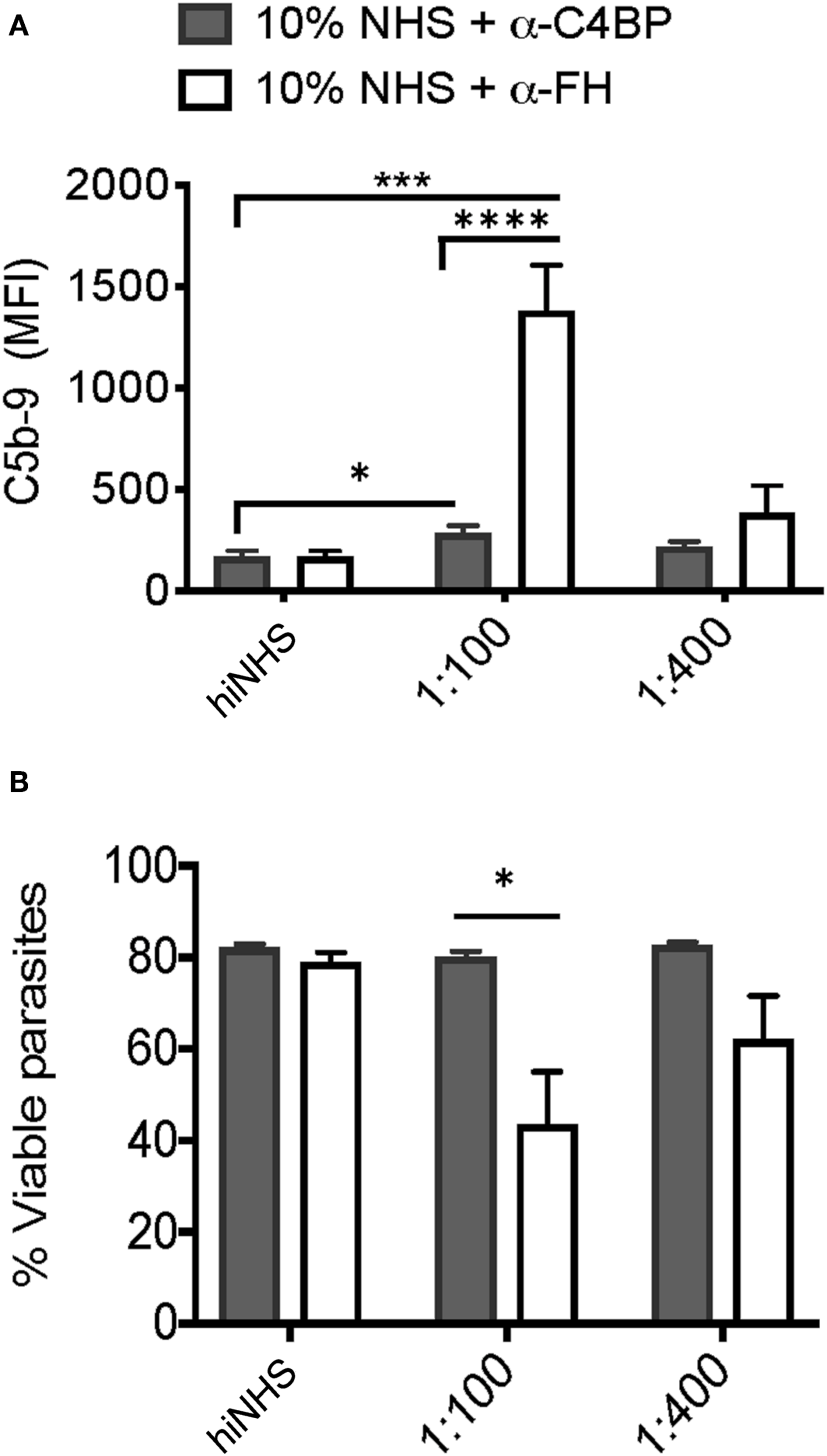
Factor H and C4b-binding protein contribute to serum resistance. Factor H (FH) and C4b-binding protein (C4BP) were blocked by pre-incubating 10% NHS with 1:100 or 1:400 dilution of goat α-human FH (CompTech) or rabbit α-human C4BP (AssayPro) for 1 h on ice before adding to 1 × 10^6^ parasites and incubating for 60 min at 37°C. Flow cytometric analysis of C5b-9 formation **(A)** and parasite viability **(B)** after 60' in 10% NHS blocked with 1:100 or 1:400 of α-C4BP (gray bars) α-FH (open bars) antibodies. 10% heat inactivated serum (hiNHS) was used a negative control. Flow cytometry data are shown as mean ± SEM from three independently performed experiments. Significant differences between the compared groups was determined using multiple Student's *t* test with Holm-Sidak correction for multiple comparisons, *p < 0.05, ***p < 0.001, ****p < 0.0001.

Parasites often target multiple complement pathways to achieve complement resistance (41). Both C4BP and FH contribute to protection against complement-mediated lysis by inactivating the classical/lectin and alternative pathways, respectively. To test the relative and contributing roles of Factor H and C4BP in blocking complement mediated killing at the parasite surface, Factor H and C4BP activity was blocked in a titratable manner using polyclonal Factor H or C4BP antibodies. A significant increase in C5b-9 formation was observed when either C4BP or FH was blocked (Figure 6A) however, decreased viability (Figure 6B) was only observed after blocking FH, and not C4BP. These results may indicate that parasite recruitment of Factor H and regulation of the alternative pathway is more relevant to confer serum resistance in *Toxoplasma*. However, it cannot be ruled out that C4BP, a large spider-like molecule consisting of 7 alpha chains (42) was more refractory to antibody blocking, such that it failed to reach the threshold of C5b-9 complex formation to affect parasite viability. Furthermore, whether C4BP and FH, other potential inhibitors, cooperate to regulate complement deposition and activation state was not investigated and requires further investigation.

### C3 Deficient Mice Have High Parasite Burdens and Reduced IgG Production and Are More Susceptible to Acute *T. gondii* Infection

**Figure 7:**
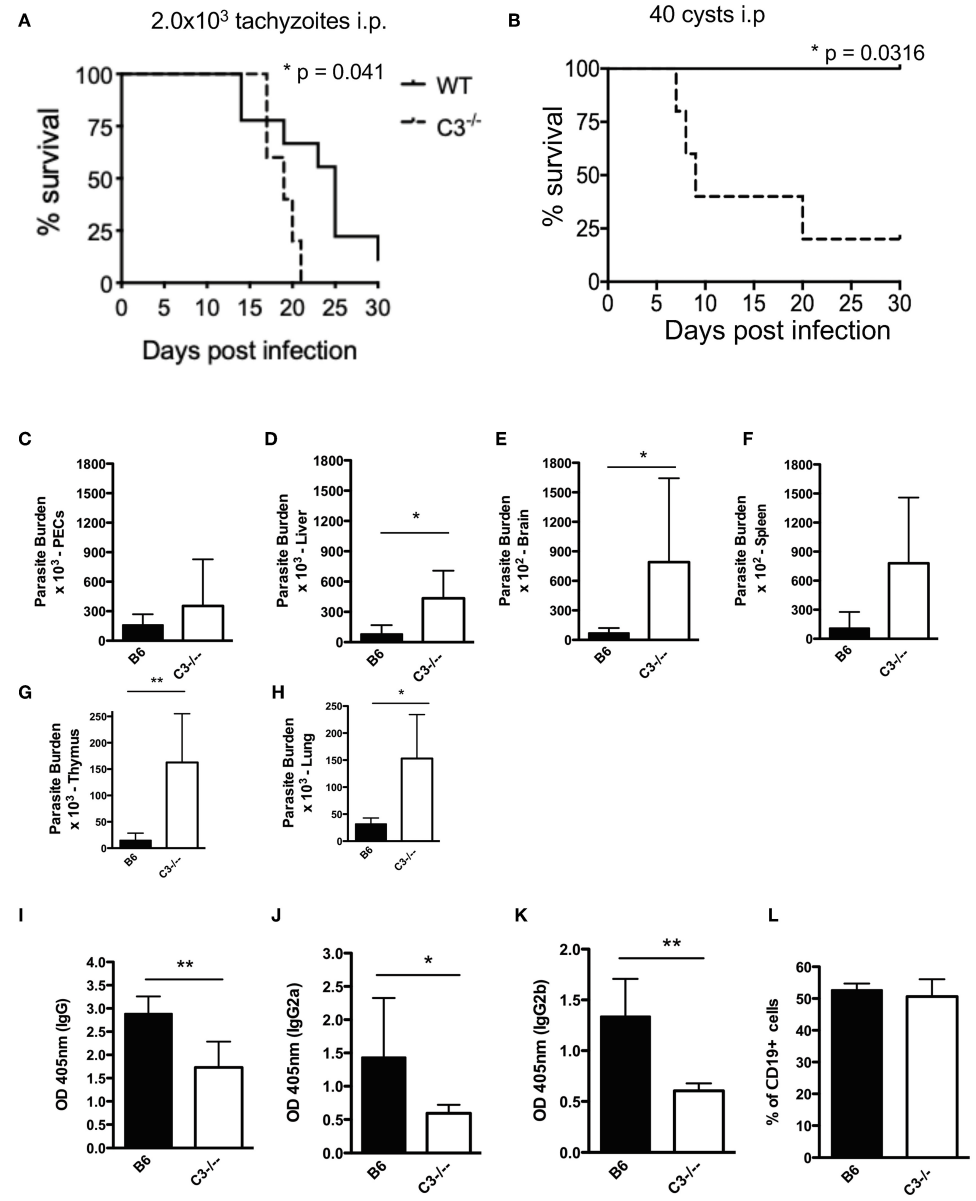
C3 contributes to protection against acute *T. gondii* infection. Survival of 6–8 week old C57BL6 (*n* = 10) and C3^−/−^ (*n* = 6) female mice infected with **(A)** 2.0 × 10^3^ CZ1 tachyzoites intraperitoneally or **(B)** 40 ME49 cysts intraperitoneally. Survival rates were compared by log-rank survival analysis of Kaplan-Meier curves, *p* = 0.041 and *p* = 0.0316. Mice infected with 40 cysts intraperitoneally were sacrificed 7 days post infection (*n* = 5). Parasite burdens were determined by counting plaque forming units (PFUs) by plating **(C)** peritoneal fluid, and homogenates of **(D)** liver, **(E)** brain, **(F)** spleen, **(G)** thymus, and **(H)** lungs onto HFF monolayers in 12 well-plates. Total levels of IgG **(I)**, IgG2a **(J)**, and IgG2b **(K)** 7 days post infection with 40 cysts i.p. were measured by ELISA. **(L)** Percentage of CD19+ splenocytes in wild type (B6) and C3^−/−^ mice. Data shown as mean ± SEM from one representative of two independently performed experiments. Significant differences between the compared groups was determined using unpaired Student's *t* test, *p < 0.05, **p < 0.01.

Complement is traditionally viewed as an innate effector system that activates directly on pathogen surfaces to facilitate their rapid lysis. All pathogens with extracellular phases in their life cycle need to regulate the complement system in order to establish infection. To establish that complement protein C3 is protective during acute infection *in vivo*, C57BL/6J mice and C3 deficient mice were infected with either Type II tachyzoites (Figure 7A) or tissue cysts (Figure 7B) intraperitoneally. To confirm this phenotype, F2 C57BL/6J C3^−/−^ homozygous and F2 heterozygous mice, which were generated by mating F1 progeny from a C57BL/6J × C3^−/−^ cross with a homozygous C3^−/−^ mouse, were also infected with tissues cysts intraperitoneally (Supplemental Figure 4). All C3 deficient mice infected with tachyzoites or tissue cysts were more susceptible to infection (Figures 7A,B and Supplemental Figure 4), the result of unregulated parasite proliferation. Parasite burdens assessed at day 7 post infection demonstrated that most tissues had increased parasite burdens and that parasites had disseminated more rapidly throughout C3 deficient mice compared to wild type animals (Figures 7C–H). Because C3 deficient mice are known to have impaired antibody responses following microbial challenge (43–45), IgG responses were evaluated in response to acute *T. gondii* infection. A general reduction in the levels of IgG were observed (Figure 7I), with significant decreases in subclasses IgG2a and IgG2b (Figures 7J,K), however there were no changes in the percentage of B cells between the groups of mice (Figure 7L). These results establish that C3 is host protective, that it limits parasite proliferation and makes mice resistant to acute infection. However, the ability of *Toxoplasma* parasites to prevent C5b-9 complex formation also renders mice susceptible to chronic infection, with all surviving mice developing infectious, transmissible tissue cysts capable of perpetuating the parasite life cycle.

## Discussion

*Toxoplasma gondii* is known to regulate several aspects of innate immunity to control immune outcome in favor of parasite persistence and their increased transmissibility to new hosts. One major effector system of innate immunity that all pathogens must overcome is the complement system, which can directly lyse pathogens upon activation. Many intracellular pathogens are resistant to lysis by employing several strategies to inactivate complement. In recent years, the role of complement during infection and disease has expanded beyond the traditional effector functions of direct lysis and has been shown to regulate both B cell and T cell immunity (45–47). In light of these new insights, many pathogens have been shown to exploit their ability to regulate complement in order to take advantage of its link to adaptive immunity (46). However, since the initial study done 30 years ago, the mechanism by which *T. gondii* regulates complement inactivation and the biological relevance of complement *in vivo* remains unknown.

In this study, we used a combination of flow cytometric assays and western blotting to investigate the mechanism of C3b inactivation *in vitro* and evaluated the impact of complement evasion on host defenses *in vivo*. The significance of our findings is 2-fold. First, we determined that *T. gondii* exploits host regulators by recruiting C4BP and FH to the parasite surface to prevent C5b-9 formation. FH specifically protected parasites from complement-mediated lysis in non-immune human serum. Second, despite the parasite's ability to inactivate C3b, *in vivo* studies using C3 deficient mice established that C3 is a critical factor necessary to regulate parasite proliferation and dissemination and promote a chronic, transmissible infection. These results are in accordance with previous studies that have similarly shown that deficiencies in components of humoral immunity, including B cells and IgM, during acute *T. gondii* infection likewise resulted in early mortality (48) and higher parasite burdens (48, 49), establishing that humoral immunity has a role in controlling acute disease (48, 50). This is the first evidence demonstrating the importance of the complement system in resistance to acute *T. gondii* infection and provides insight into the critical balance that the parasite must strike mediating both immune activation as well as immune regulation to influence an immune outcome that promotes parasite transmissibility.

There were two findings in this study that are at odds with the initial study done 30 years ago. First, we showed that Type I and Type II strains exhibited differential C3b deposition. Second, despite using the same concentration of serum and a similar approach using MgEGTA to determine the contribution of the alternative pathway to C3b deposition, we showed that Ca^2+^ dependent lectin pathway activation, and not the alternative pathway, was the major contributor initiating C3b deposition. We suspect that the discrepancies in our findings may be attributed to the differences in the experimental approach and the parasites strains used. Here, we used specific monoclonal antibodies to detect C3b, whereas the previous approach supplemented NHS with radiolabeled C3. It is possible that using specific antibodies to detect C3b was a more sensitive assay. Second, a majority of our analyses were done using a Type II strain, whereas the previous study primarily used a Type I strain, which we showed to possess relatively low levels of C3b on their surfaces which may have impacted the sensitivity of the previous assay. Here, additional evidence further supported a role for lectin pathway activation on the *T. gondii* surface. We used flow cytometric lectin binding assays to show differences in the levels and heterogeneity of surface glycans between Type I and Type II strains. Importantly, this heterogeneity correlated with differences in parasite recognition by MBL. Furthermore, MBL deficient serum exhibited a decrease in C3b deposition on Type II strains. Although recent studies have shown that the upregulation of cerebral C1q expression during chronic *T. gondii* infection (51) suggests that the classical pathway plays a role during chronic infection, we did not find evidence of C1q binding directly to the tachyzoite surface in non-immune serum (Supplemental Figure 3).

The presence of both C4BP and FH on the parasite surface suggests that *T. gondii* regulates all complement pathways. However, when we blocked C4BP or FH to determine their relative contributions to parasite resistance to serum killing, we found it surprising that C4BP did not significantly contribute to parasite resistance to lysis considering the contribution of the lectin pathway in C3b deposition. Because we assayed for C3b levels and activation state, it is possible that C4BP was more important in the regulation of C4b than C3b, as has been demonstrated previously (39). Alternatively, as a large spider-like molecule, it is possible that the polyclonal antibody used to block C4BP was inefficient and failed to completely block C4BP, thus depleted sera should be used to better assess the contribution of C4BP to complement regulation.

The ability of FH to protect parasites from lysis in this study supports an important role for regulation of the AP on the parasite surface. In fact, the role of AP activation has been shown to be more critical downstream of classical and lectin pathway activation, where AP effectively utilizes C3b deposited by classical or lectin pathways as a platform for generating AP convertases to rapidly generate more C3b, also known as the alternative pathway amplification loop, and thus the AP is quantitatively responsible for up to ~80% of C5b-9 formation (52–54). *Toxoplasma gondii* is known to be susceptible to classical pathway activation in the presence of IgG and IgM (30, 55). The Sabin-Feldman dye test is a diagnostic test which relies on live parasites and parasite susceptibility to the classical pathway to diagnose *T. gondii* infection in humans. Early studies investigating the cytolytic mechanisms behind the Sabin-Feldman dye test provided evidence for the requirement of the AP for parasite lysis to occur, however these results conflicted with several other studies (30, 56, 57). In this context, the rapid amplification of AP may exist as the more relevant pathway the parasite must inactivate to resist serum killing in non-immune human serum.

The capacity of *T. gondii* to recruit C4BP and FH to its surface raises an interesting set of questions pertaining to exactly how the parasite achieves this and what parasite factors interact with these regulators. Recent studies have shown that *Plasmodium falciparum* recruits FH via surface protein Pf92 (13), a member of the stage-specific *Plasmodium* surface proteins known as the 6-CYS proteins. Previous comparative modeling work suggested that the 6-CYS superfamily of 14 proteins encoded by *Plasmodium* share a structural fold in similar with a family of *T. gondii* proteins that dominate the parasite surface, known as the SRS (SAG-1 related sequences) proteins (58). This was later confirmed when the crystal structure was solved for the *Plasmodium* 6-CYS protein Pf12, revealing a tertiary structural homology with the SRS domain fold (59–61). This evidence for complement evasion mediated by the *Plasmodium* 6-CYS proteins establish a rationale to investigate whether structurally homologous *Toxoplasma* SRS proteins likewise regulate the complement system during active infection. The mechanism by which Pf92 recruits FH, however, is unknown. It is well-established that FH is recruited to host cells by recognizing C3b and ubiquitous host polyanion structures like sialic acid or heparan sulfated proteoglycans (SPGs) (62, 63). Some bacterial pathogens exploit this interaction by either scavenging sialic acid to coat their surfaces or synthesizing their own to recruit FH and thereby protect their surface from complement attack (64–66). Several studies have demonstrated that *T. gondii* possess a lectin like activity associated with its ability to interact with host SPGs (67, 68) and sialic acid (69, 70). It has been reported that C4BP also contains a binding domain for heparin (63). Intriguingly, the crystal structure for surface protein SRS29B revealed a dimer dependent basic groove capable of docking a host heparan sulfated proteoglycan (59). Atomic resolution studies of the microneme protein MIC1 has also identified a novel cell binding motif that is associated with binding sialylated structures (71). It is possible that *T. gondii* utilizes its capacity to bind SPGs and sialic acid as a mechanism for FH and C4BP recruitment. Comparative modeling and *in vitro* studies suggested that SRS57 (formerly SAG3) binds SPGs, thus it is a strong candidate for future studies (59, 72). Thus, identification of the parasite factors that mediate FH and C4BP recruitment would further our understanding of these interactions.

We observed heterogeneity in surface glycans between Type I and Type II strains that was associated with differences in MBL recognition, suggesting differences in surface protein composition between strains may impact complement activation and C3b deposition. Our lab has recently identified differential expression of SRS genes in a developmental-stage- and strain-specific fashion (73). Thus, the SRS superfamily of proteins represent strong candidates to pursue as MBL and C3 acceptor molecules. Furthermore, this strain specific difference in C3b deposition is a mappable trait, and forward genetics studies are underway to identify the parasite factors controlling this trait. However, despite the differences in complement activation between Type I and Type II strains, parasites were equally resistant to serum killing, by their ability to inactivate surface bound C3b and thus prevent the formation of C5b-9. These differences raise an interesting the question about the biological significance of the level of C3b and if these differences impact C3-dependent effector functions, such as phagocytosis and stimulation of B cell immunity. While many pathogens have efficient strategies to resist complement-mediated killing in order to disseminate or persist throughout infected host, the complement system has overcome this step by employing several protective effector functions that are dependent on the generation of C3b and C3a. In this study, intraperitoneal infection of C3 deficient mice determined that C3 was protective *in vivo*. However, one limitation in using these mice is that we cannot distinguish the relative and contributing roles of C3b vs. C3a in the induction of sufficient protective immunity capable of regulating parasite proliferation.

It is highly likely that the parasite's ability to regulate the level and form of C3b may be used to exploit other effector functions that modulate virulence. In this study, we determined that the surface of *T. gondii* is predominantly coated with iC3b and C3dg, which are important effector molecules that are ligands for several receptors on both myeloid cells and lymphocytes, thus having potentially important implications in pathogenesis. Our study does provide some evidence to suggest that, like several pathogens, *T. gondii* may exploit complement and its link to adaptive immunity to control immune outcome. Given the role of adaptive immunity in controlling *T. gondii* proliferation, the decrease in IgG production in C3^−/−^ mice indicates that covalent modification of surface antigen with C3dg may influence B cell immunity. Alternatively, opsonization with iC3b have been associated with increased phagocytosis for several pathogens (74, 75). Although phagocytosis is not the major mode of *T. gondii* entry into the cell, recent studies have shown that mouse avirulent Type II strains are preferentially phagocytosed by murine macrophages, whereas mouse virulent Type I strains are more invasive (76). Thus, future work will be necessary to determine specifically how complement regulation affects these critical effector functions.

To our knowledge, this is the first report demonstrating that *T. gondii* recruits host regulators to mediate serum resistance. Furthermore, we establish that C3 is an important factor regulating parasite proliferation and antibody responses *in vivo*. Taken together, our findings provide critical insight into the mechanisms by which *Toxoplasma gondii* strikes a critical balance *in vivo* by inactivating complement to protect the parasite from serum killing, while capitalizing on its important link to stimulate sufficient protective immunity to regulate tachyzoite proliferation and promote host survival, parasite persistence, and transmissibility to new hosts.

## Data Availability Statement

The datasets generated for this study are available on request to the corresponding author.

## Ethics Statement

The animal study protocol LPD 22E was reviewed and approved by the Animal Care and Use Committee of the Intramural Research Program of the National Institute of Allergy and Infectious Diseases, National Institutes of Health.

## Author Contributions

Research idea and study design was by MG, PS, and AC. PS performed *in vitro* and *in vivo* experiments, performed analysis and interpretation of the data, and wrote the paper. AC performed i.p. experiments with cysts and performed data analysis. MG supervised the development of work, data interpretation and helped write and edit the manuscript.

### Conflict of Interest

The authors declare that the research was conducted in the absence of any commercial or financial relationships that could be construed as a potential conflict of interest.

## Supplementary Material

Supplemental Figure 1 *Toxoplasma gondii* binds C3b. **(A)** Flow cytometric analysis of complement activation by measuring C3b deposition on Type II CZ1 parasites incubated in 10% NHS in HBSS^++^ for 30 min at 37°C. Parasites were gated on the basis of forward and side scatter. **(B)** Representative histograms of C3b deposition on Type II parasites in 10% NHS (solid black line) and heat inactivated NHS (hiNHS, solid gray shaded) in HBSS^++^ for 30 min at 37°C. Unstained parasites were used a negative control (black dotted line).

Supplemental Figure 2 Type I and Type II parasites inactivate C3b in a time dependent manner. Western blot analysis comparing the kinetics of C3b inactivation on 5 × 10e5 Type II CZ1 parasites **(A)** and Type I RH parasites **(B)** over 60 min in 10% NHS. Blots were probed for C3 and its catabolites using a goat α-human C3 polyclonal antibody 1:20,000 (CompTech) and donkey α-goat 1:5,000 (Santa Cruz Biotechnology). Arrows correspond to sizes of C3 (active C3b α chain 110 kDa, β chain 75 kDa; inactive iC3b α_1_ 68 & α_2_ 43 kDa, and C3dg 41 kDa). SRS29B (formerly SAG1, 1:5,000) was used as loading control.

Supplemental Figure 3 C1q does not bind *T. gondii* in non-immune serum. Representative histogram of flow cytometric analysis of C1q binding to Type II parasites in non-immune and immune serum. CZ1 Type II parasites were incubated in 10% non-immune serum (black solid line) or 10% immune serum (gray solid line) for 10 min and stained with a monoclonal anti-human C1q antibody (Cedarlane, 1:250). Secondary stain alone was used a negative control (dotted black line).

Supplemental Figure 4 Survival kinetics of 6–8 week old F2 homozygous C57BL/6J C3^−/−^ mice (*n* = 8) vs. F2 heterozygous mice (*n* = 5), generated by mating F1 progeny from a C57BL/6J WT x C3^−/−^ mouse cross with C3^−/−^ homozygous mice, infected interperitoneally with 35 ME49 cysts. Survival rates were compared by log-rank survival analysis of Kaplan-Meier curves, *p* = 0.0367.
