# Supplementary figures and images for: Toxoplasma gondii Recruits Factor H and C4b-Binding Protein to Mediate Resistance to Serum Killing and Promote Parasite Persistence in vivo
Source: Front Immunol. 2020 Jan 17;10:3105. doi: 10.3389/fimmu.2019.03105 (PMC6979546; doi:10.3389/fimmu.2019.03105)

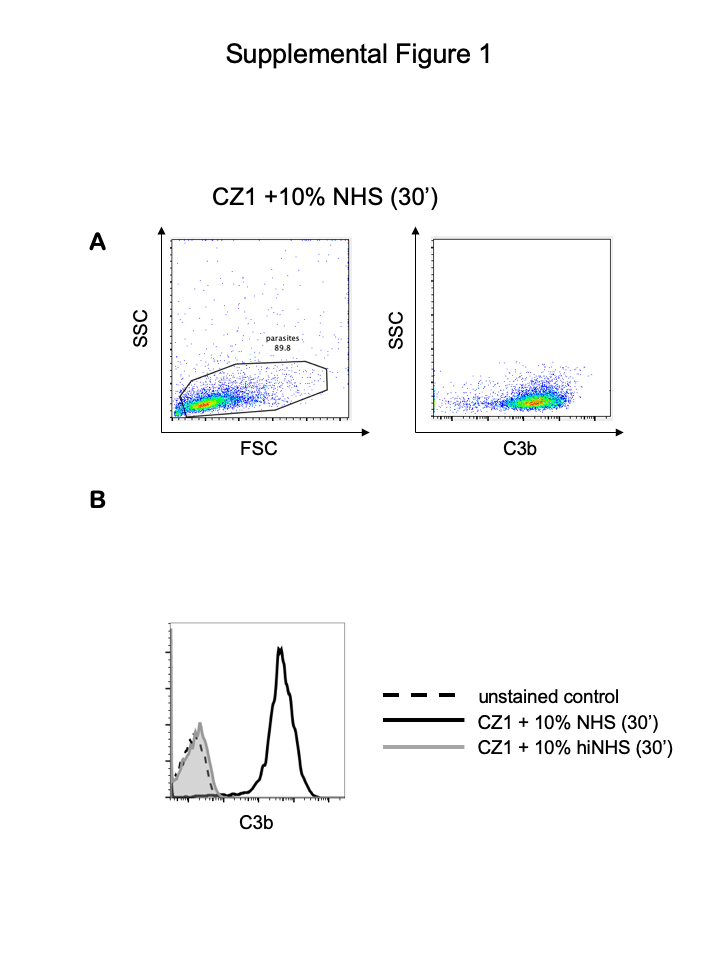

Supplement: Supplemental Figure 1 — Toxoplasma gondii binds C3b. (A) Flow cytometric analysis of complement activation by measuring C3b deposition on Type II CZ1 parasites incubated in 10% NHS in HBSS++ for 30 min at 37°C. Parasites were gated on the basis of forward and side scatter. (B) Representative histograms of C3b deposition on Type II parasites in 10% NHS (solid black line) and heat inactivated NHS (hiNHS, solid gray shaded) in HBSS++ for 30 min at 37°C. Unstained parasites were used a negative control (black dotted line). [file Image_1.TIFF]

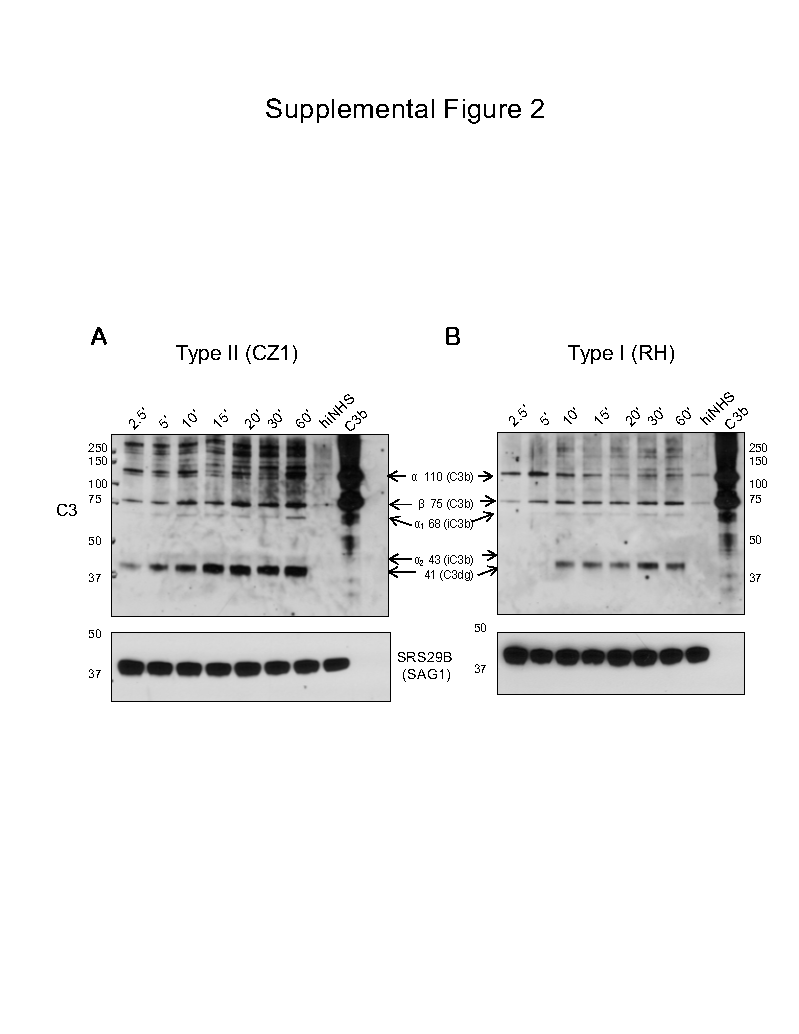

Supplement: Supplemental Figure 2 — Type I and Type II parasites inactivate C3b in a time dependent manner. Western blot analysis comparing the kinetics of C3b inactivation on 5 × 10e5 Type II CZ1 parasites (A) and Type I RH parasites (B) over 60 min in 10% NHS. Blots were probed for C3 and its catabolites using a goat α-human C3 polyclonal antibody 1:20,000 (CompTech) and donkey α-goat 1:5,000 (Santa Cruz Biotechnology). Arrows correspond to sizes of C3 (active C3b α chain 110 kDa, β chain 75 kDa; inactive iC3b α1 68 & α2 43 kDa, and C3dg 41 kDa). SRS29B (formerly SAG1, 1:5,000) was used as loading control. [file Image_2.TIFF]

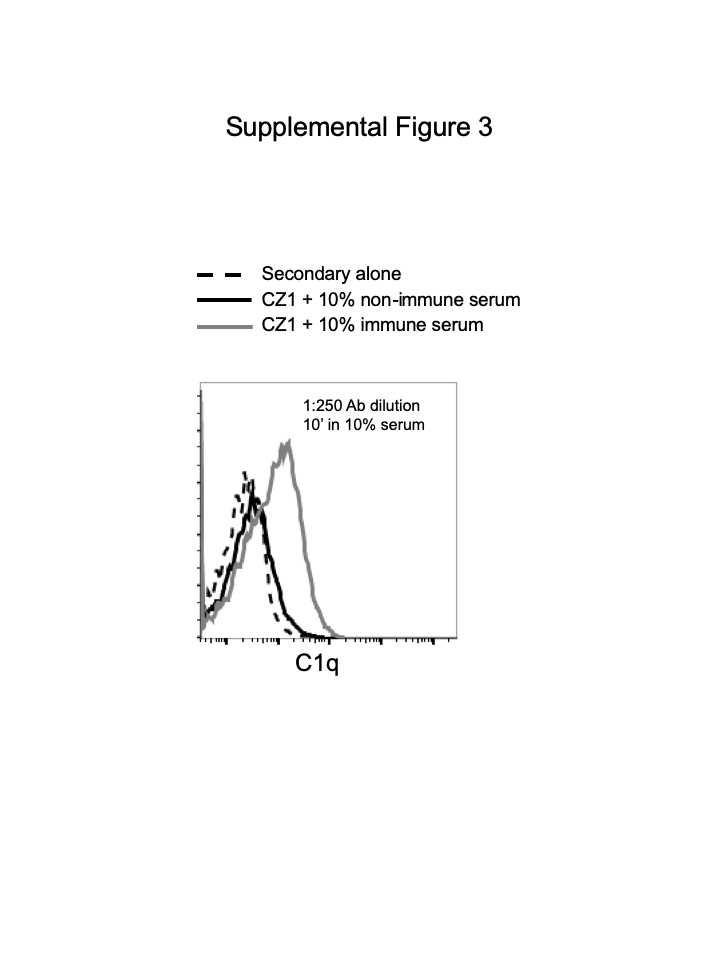

Supplement: Supplemental Figure 3 — C1q does not bind T. gondii in non-immune serum. Representative histogram of flow cytometric analysis of C1q binding to Type II parasites in non-immune and immune serum. CZ1 Type II parasites were incubated in 10% non-immune serum (black solid line) or 10% immune serum (gray solid line) for 10 min and stained with a monoclonal anti-human C1q antibody (Cedarlane, 1:250). Secondary stain alone was used a negative control (dotted black line). [file Image_3.TIFF]
